# The Challenges of Performing Exome Sequencing in Structurally Normal Fetuses

**DOI:** 10.1002/pd.6687

**Published:** 2024-10-11

**Authors:** Natalie Chandler, Muriel Holder‐Espinasse, Fionnuala Mone

**Affiliations:** ^1^ North Thames Genomic Laboratory Hub Great Ormond Street Hospital for Children NHS Foundation Trust London UK; ^2^ Clinical Genetics Department Guy's and St Thomas' NHS Foundation Trust London UK; ^3^ Centre for Public Health Queen's University Belfast Belfast UK

**Summary**

- What’s already known about this topic?
  ◦ Exome sequencing in structurally normal fetuses is not currently representatative of wider routine clinical practice internationally
- What does this study add?
  ◦ This summates recent evidence regarding studies assessing the clinical utility of prenatal exome sequencing in structurally normal fetuses
  ◦ This highlights the interpretational and ethical challenges posed by performing prenatal sequencing in this scenario

Since the advent of prenatal exome sequencing (pES), it has typically only been offered and performed for fetuses where a structural anomaly has been suspected prenatally. In this setting there is already a wealth of evidence to support a significant incremental diagnostic yield over standard testing strategies [1] There is a recent small body of research assessing its application in fetuses with no identifiable phenotype, or so‐called “structurally normal fetuses.” While we value the importance of collecting data pertaining to the clinical utility in this group, results must be interpreted with caution.

One of the first studies in this area was reported by Vaknin et al. where 160 “structurally normal fetuses” underwent pES [2]. Of these, 1 (0.6%) was found to have an “early onset severe genetic disorder” which was due to a *de novo* heterozygous variant in the *KCNK4* gene causative of *Facial dysmorphism, hypertrichosis, epilepsy, intellectual/developmental delay and gingival overgrowth syndrome* (Table 1). The pregnancy was terminated. However, and as noted in OMIM, regarding *KCNK4*, of the three cases reported thus far “developmental progress was highly variable” [3]. The study concludes that “these results show a high rate of abnormal findings on pES even in apparently normal pregnancies.” While it could be anticipated that one case of 160 structurally normal fetuses could have a disease causing genomic variant, one case is too few to support such a conclusion [2].

**TABLE 1:** Pathogenic and likely pathogenic variants causative of “severe phenotypes” in structurally normal fetuses.

Study	Gene	Genotype	ACMG classification	Syndrome (OMIM)	Evidence	Zygosity
Vakin et al. 2022	*KCNK4*	c.698C>T p.Pro233Leu	P	Facial dysmorphism, hypertrichosis, epilepsy, intellectualintellectmental delay and gingival overgrowth syndrome (618381)	PM2, PS4, PS2	Het (*de novo*)
Daum et al. 2023	*ATP7B*	c.3191A>C p.(Glu1064Ala)	P	Wilson disease (277900)	PM1, PM2, PP3, PP5, PM5	Comp het (mat)
		c.3451C>T p.(Arg1151Cys)	LP		PM1, PM2, PM5, PP3	Comp het (pat)
	*SPRED1*	c.349C>T	P	Legius syndrome (611431)	PM2, PVS1, PP5, PP3, PS2	Het (*de novo*)
		p.(Arg117Ter)				
Levy et al. 2024	*ADNP*	c.1223dupA	LP	Helsmoortel‐van der As syndrome: ADNP‐related multiple congenital anomalies‐intellectual disability‐Autism spectrum disorder (615873)	PM2, PVS1	Het (*de novo*)
		p.Ser409fs				
	*SETD1A*	c.757C>T	LP	Neurodevelopmental disorders with speech impairment and dysmorphic facies (619056)	PVS1, PM2	Het (*de novo*)
		p.Arg253*				
	*PUM1*	c.2173delA	LP	Spinocerebellar ataxia 47 (617931)	PVS1, PM2	Het (*de novo*)
		p.Thr725fs				
	*ANKRD11*	c.2408_2412delAAAAA	P	KBG syndrome (148050)	PVS1, PS4, PM2	Het (*de novo*)
		p.Lys803fs				
	*TSC2*	c.3922_3923delGT	LP	Tuberous sclerosis‐2 (613254)	PVS1, PM2	Het (*de novo*)
		p.Val1308fs				
	*CUL3*	c.1664dupG	LP	Neurodevelopmental disorder with or without autism or seizures (619239)	PVS1, PM2	Het (*de novo*)
		p.Ser556fs				
	*SCN8A*	c.1089A>T	LP	Neurodevelopmental disorder and structural brain anomalies with or without seizures and spasticity (614558)	PM1, PM2, PM5, PP2, PP3	Het (*de novo*)
		p.Leu363Phe				
	*WAC*	c.1477_1480del	LP	Desanto‐Shiwani syndrome (616708)	PVS1, PM2	Het (*de novo*)
		p.Ser493ProfsTer6				
	*KAT6A*	c.4460_4463delinsGTG	LP	Arboleda‐Tham syndrome (616268)	PVS1, PM2	Het (*de novo*)
		p.Gln1487ArgfsTer46				

Abbreviations: ACMG, American College of Medical Genetics and Genomics; comp het, compound heterozygous; het, heterozygous; LP, likely pathogenic; P, pathogenic.

Another study from Daum et al. reported a diagnostic yield of 0.83% (*n* = 4/482) in fetuses deemed “sonographically normal” at the time of testing [4]. Two of these were classified as “severe” disease (0.41%), one being *Wilson disease* due to a homozygous *ATP7B* variant and the other *Legius (Neurofibromatosis Type 1‐like) syndrome* due to a *de novo* pathogenic variant in *SPRED1* with severity based upon a pilot study by Lazarin et al. (Table 1) [5]. Both of these cases ended in termination of pregnancy (TOP). This study classified severity based on evaluation of expanded carrier screening panels via expert consensus. Of those consulted to partake in the consensus there was a 6.4% response rate, of which over two thirds were genetic counselors [5]. In addition, the study by Daum et al. uncovered two secondary findings. Secondary findings refer to variants in disease‐causing genes that are unrelated to the phenotype, but that are actively sought. Ethically the seeking and reporting of such secondary findings poses challenges in that they can uncover a treatable or untreatable adult onset condition in a fetus, raising concerns around autonomy and justice as well as the possible impact on the subsequent child's open future. There is also potential to uncover disease associated genes such as cancer susceptibility in the parents, when this was never meant to be the purpose of the test, and this could be associated with psychological harm [6]. Given that none of the fetuses reported by Daum et al. have a demonstrable phenotype, distinguishing which variants are secondary findings or primary disease causing variants is problematic. Given there is debate and as yet no robust framework for the reporting of secondary findings specific to prenatal samples where sequencing has been initiated when a genetic syndrome has been suspected, there is further uncertainty when this is applied when such a syndrome is not phenotypically suspected [6]. Daum et al. do not disclose phenotypes for the remainder of the pregnancy or postnatally. The authors noted the potential for bias due to a high prevalence of founder mutations within the local population [3].

A further study from Levy et al. reported findings from pES in 1020 “low‐risk” cases and reported that 2.7% had potentially significant variants [7]. Again, the study from Lazarin, et al. was used to classify the severity of the syndromes identified [5]. For cases judged as having a “severe” phenotype, there were 9 (0.88%) (Table 1). The authors noted that TOP was elected in 13 out of 1020 cases (1.2%) in the cohort, including 7/9 (77.8%) in the “severe” phenotypes group. Despite being stated from study inception, that variants of uncertain significance (VUS) would not be reported, seven cases were included within the 2.7% yield which were referred to as “*hot VUS,*” hence were not diagnostic variants. Levy et al. define a “*hot VUS*” as “*being characterized by a substantial body of supporting evidence, and if further confirmation of their pathogenicity can be acquired, they might be reclassified as pathogenic.*” [7] Of these, over half ended in TOP and in case 27, the variant reported has since been removed from the carrier screening panel and the gnomADv4 population database now shows that this variant has been seen homozygously in multiple individuals indicating it is likely to be benign. As in the previous studies, no evolving phenotypic data pre‐ or postnatally was provided that is, no post‐mortem analysis after TOP, no pediatric follow‐up, no additional investigations when variants identified to support diagnoses were made and no phenotypic evaluation of parents when variants were inherited.

Since the original publication of the Richards, et al. American College of Medical Genetics and Genomics (ACMG) variant classification guidelines, there have been a number of separate articles from the ClinGen sequence variant interpretation group in relation to specific individual criteria aimed at improving consistency and transparency in classification rationale [8, 9]. However, there is currently no overarching article combining all of the specific criteria recommendations for general variant classification although there are many disease specific ones. Without such an article, it results in variability as to which of these separate articles are being applied. In the study from Levy et al. it is noted that ClinGen recommendations for the use of *in silico* tools for PP3 and BP4 were not applied in their variant classification and that the guidance on use of absence/low frequency of the variant in population databases (PM2) at supporting level was applied [10, 11]. The reason for these choices is not stated. The authors of this study also do not indicate what *in silico* tools were used when applying PP3 criteria. Upon review of the variants, other specific guidelines such as the one for PVS1, which is critical in the classification of premature truncating variants, do not appear to have been applied [12]. The UK Association for Clinical Genomic Science (ACGS) have published an overarching document which is updated annually. This document also provides further guidance on the application of other criteria that have not been addressed by the ClinGen team as of yet such as PM1‐ “located in a mutational hotspot without benign variation” and PS4—“the prevalence of the variant in affected individuals is significantly increased compared with the prevalence in controls” [13].

On independent re‐analysis of the variants in group 1 of Levy et al. (i.e., *de novo* pathogenic and likely pathogenic variants in genes causing “severe phenotypes” in “low‐risk” cases) using the UK ACGS guidelines, over half of the reported nine pathogenic/likely pathogenic variants could be reclassified as VUS. Reasons for the potential over‐classification included differences in the guidance for variant classification being applied. Patients may decide to terminate based on such variants and in the absence of follow up, some of these may have been normal fetuses. This highlights the requirement for variant classifications to be reviewed during the peer review process and for future guidelines to provide clarity and uniformity regarding variant classification to try to address this variability, particularly in relation to variants in rare genes. It is important that there is transparency provided regarding variant interpretation and that variants are reported within public databases so outcomes of pregnancies that have undergone sequencing in the setting of an apparently normal pregnancy can be collated.

When performing pES in structurally normal fetuses challenges lie not only in variant interpretation but also in clinical interpretation. Even if a variant is reported as pathogenic or likely pathogenic this does not always equate to a clinical diagnosis [14]. If the prior probability of a genetic disease is extremely low within the population, in the absence of a family history or a demonstrable phenotype, the likelihood that the fetus is affected remains low [14]. Many variants reported in the aforementioned three studies are associated with clinical variability and incomplete penetrance, hence one must ask if these are indeed actionable findings in that whether they fit criteria for TOP. Of note, all of the aforementioned evidence is from one country, Israel, which is unique in that healthcare professionals are legally obligated to inform pregnant patients of all globally available genetic tests, regardless of the indication. This means that patients may prefer to opt for invasive advanced genomic testing more frequently than in other countries. In addition, there is no gestational age upper limit for TOP, if the risk of a “severe medical condition” is greater than 30%. Moreover, ethnic adjusted carrier screening is performed routinely from the outset. One must therefore question the personal utility of findings internationally given these legal, economical, ethical and cultural differences. Given that Daum et al. and Levy et al. are reporting likely pathogenic (class IV) variants as disease causing (Table 1), with a 10% chance of these not being pathogenic, for disorders that are rare in the population, or with variable penetrance, this compounds the uncertainty about whether the fetus would be affected [8]. Bayesian frameworks are applied within the ACMG classification of variants through combining population, functional and segregation data to predict the probability of disease and the commonly known stratified classification system of variants from benign to pathogenic [8]. Similar applications have been shown to estimate variant induced disease risk and the probability of expression of disease where penetrance may cause variable phenotypic impact. An area where such application could be considered is for fetuses with no ultrasound findings where a variant has been identified and the probability of an impact of that variant in childhood or adulthood could potentially therefore be predicted [15].

Additionally, as alluded to by one of the authors there is the issue of potential incomplete phenotyping in that “*many ultrasound findings either can't be seen during the pregnancy or findings may show later during the pregnancy*” [2]. Is it possible the fetuses were not structurally normal in the first instance and that this was missed? This may have been the situation in case #5 (Tuberous sclerosis) where deeper phenotyping using fetal echocardiogram by a pediatric cardiologist or a fetal whole body MRI may have unveiled a phenotypic finding such as a tuber. The accuracy of fetal anatomical ultrasound specific to the population or associated phenotypes later in pregnancy have not been reported so it is unknown If the fetuses truly are “structurally normal.” Some phenotypes only evolve over time for example fetal growth restriction and may only become evident very late in the pregnancy if at all for example fetal brain anomalies [16]. By not following up cases and reporting phenotypic evolution if apparent, stating that genomic diagnoses have been made in “structurally normal” fetuses may be misleading.

The performance of advanced genomic testing in structurally normal fetuses raises ethical challenges, most notably that of beneficence versus non‐maleficence for health care professionals. Exome sequencing is an expensive test; can a screening test with such a yield be a cost‐effective strategy? The authors of the previous studies state that knowledge of a diagnosis can guide in‐utero therapies, however at present there are no in‐utero therapies for any of the genetic syndromes detected. A further justification for testing is that it is not a dissimilar strategy to the ACMG recommendation regarding the reporting of secondary findings in that highly penetrant pathogenic variants unrelated to the phenotype, and often not associated with a phenotype are reported. While this is true, the list of genes relating to this is restricted and the patients are undergoing the invasive testing in the first instance because the fetus has structural anomalies [17].

At present, pES in structurally normal fetuses is not endorsed by any international governing bodies and raises ethical concerns. It is important that clinicians interpreting research in this area deeply appraise the results and findings and that subsequent released guidance regarding variant interpretation endorses uniformity and clarity around variant classification. This should include recommendations on what variants should be reported in structurally normal fetuses, whether variants that are present in general population datasets should be reported, what clinical syndrome counts as being severe enough to be reported, how to address the issues of incomplete penetrance and variable expressivity with no phenotype. It is also unknown whether there is a lower degree of penetrance for inherited variants with no familial phenotypic expression of the disorder as historically any evidence for classifying variants has been based on testing affected patients. Authors publishing pathogenic and likely pathogenic variants have a duty to future patients with the same variants, where their evidence could impact on variant classification and potential clinical outcomes.

## Ethics Statement

The authors have nothing to report.

## Consent

The authors have nothing to report.

## Conflicts of Interest

The authors declare no conflicts of interest.

## Data Availability

The authors have nothing to report.
